# Pharmacological Evaluation of Secondary Metabolites and Their Simultaneous Determination in the Arabian Medicinal Plant *Plicosepalus curviflorus* Using HPTLC Validated Method

**DOI:** 10.1155/2019/7435909

**Published:** 2019-03-19

**Authors:** Raha Orfali, Shagufta Perveen, Nasir Ali Siddiqui, Perwez Alam, Tawfeq Abdullah Alhowiriny, Areej Mohammad Al-Taweel, Sami Al-Yahya, Fuad Ameen, Najwa Majrashi, Khulud Alluhayb, Bandar Alghanem, Hayat Shaibah, Shabana Iqrar Khan

**Affiliations:** ^1^Department of Pharmacognosy, College of Pharmacy, King Saud University, P.O. Box 2457, Riyadh 11451, Saudi Arabia; ^2^National Center for Biotechnology, Life Science and Environment Research Institute, King Abdulaziz City for Science and Technology (KACST), P.O. Box 6086, Riyadh 11461, Saudi Arabia; ^3^Department of Microbiology, College of Science, King Saud University, P.O. Box 2457, Riyadh 11451, Saudi Arabia; ^4^Medical Core Facility and Research Platforms, King Abdullah International Medical Research Center/King Saud Bin Abdulaziz University for Health Sciences, Ministry of National Guard Health Affairs, Riyadh 11426, Saudi Arabia; ^5^National Center for Natural Products Research, School of Pharmacy, University of Mississippi, Oxford 38677, USA

## Abstract

*Plicosepalus* is an important genus of the Loranthaceae family, and it is a semiparasitic plant grown in Saudi Arabia, traditionally used as a cure for diabetes and cancer in human and for increasing lactation in cattle. A flavonoid quercetin (**P1**), (-)-catechin (**P2**), and a flavane gallate 2S,3R-3,3′,4′,5,7-pentahydroxyflavane-5-O-gallate (**P3**) were isolated from the methanol extract of the aerial parts of *P. curviflorus* (PCME). The PCME and the isolated compounds were subjected to pharmacological assays to estimate peroxisome proliferator-activated receptors PPAR*α* and PPAR*γ* agonistic, anti-inflammatory, cytotoxic, and antimicrobial activities. Results proved for the first time the dual PPAR activation effect of the PCME and catechin (**P2**), in addition to the promising anti-inflammatory activity of the flavonoid quercetin (**P1**). Interestingly, both PCME and isolated compounds showed potent antioxidant activities while no antimicrobial effect against certain microbial strains had been reported from the extract and the isolated compounds. Based on the pharmacological importance of these compounds, an HPTLC validated method was developed for the simultaneous estimation of these compounds in PCME. It was found to furnish a compact and sharp band of compounds **P1**, **P2**, and **P3** at *R*_f_ = 0.34, 0.47, and 0.65, respectively, using dichloromethane, methanol, and formic acid (90 : 9.5 : 0.5, (v/v/v)) as the mobile phase. Compounds **P1**, **P2**, and **P3** were found to be 11.06, 10.9, 6.96 *μ*g/mg, respectively, in PCME. The proposed HPTLC method offers a sensitive, precise, and specific analytical tool for the quantification of quercetin, catechin, and flavane gallates in *P. curviflorus*.

## 1. Introduction

Flavonoids and flavane gallates are naturally occurring secondary metabolites in the plant species. They present in good amounts in dietary supplements and functional food. Quercetin is one of the important bioflavonoids present in many ethnic plants. Quercetin has importance in terms of ethnopharmacology due to its use as anti-inflammatory, antioxidant, anticancer, antiobesity, and antiatherosclerotic activities [1, 2].

The flavane gallates, catechins, and their derivatives have attracted attention in terms of beneficial effects on human health, due to their high availability, less toxic effect, and low cost [3]. Previous investigations on catechins reported different types of biological activities such as antitumor, antioxidative, and antimicrobial activities [4, 5].

**Figure 1:**
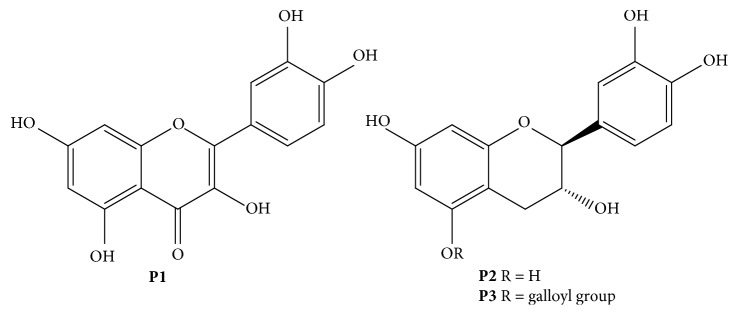
Structure of compounds **P1**, **P2**, and **P3**.

*Plicosepalus curviflorus* is one of the two known species grown in Saudi Arabia and belongs to Loranthaceae family. This parasitic plant found to be growing in East Africa, North East Africa, Saudi Arabia, and Yemen [6, 7]. In the traditional system of medicine, the stems were valued for cancer in Yemen [7, 8], while the leaves of the plant were reported for curing diabetes in Saudi Arabia [9]. Various phytochemical studies of crude leaves of *P. curviflorus* showed the presence of flavonoids, flavane gallates, sterols, and terpenoids [10]. For instance, in our previous phytochemical investigation on *P. curviflorus* growing in Saudi Arabia, we isolated the quercetin (**P1**), catechin (**P2**), and flavane gallate 2S, 3R-3,3′,4′,5,7-pentahydroxyflavane-5-O-gallate (**P3**) (Figure 1) from the aerial parts of the plant and proved their hypoglycemic effect [11].

Consequently, the present study was focused on further pharmacological investigations of the plant extract and isolated compounds in order to explore their peroxisome proliferator-activated receptors PPAR*α* and PPAR*γ* agnostic, anti-inflammatory, cytotoxic, and antimicrobial effects to verify its traditional uses and try to refer activities to the isolated compounds.

The HPTLC (high-performance thin-layer chromatography) has been widely used recently in the quality control of herbs and their formulations due to its low operation cost, high sample throughput, and small mobile phase requirement. Different wavelengths of light which observed with specific types of analyses can give a complete profile of the plant extract. It is widely used for the identification, stability, dissolution, purity testing, or content uniformity of crude extracts of the plant [12].

The good pharmacological activities shown by the methanol extract of *P. curviflorus* and its isolated compounds (**P1**, **P2**, and **P3**) motivated the authors for concurrent analysis of biomarkers **P1**, **P2**, and **P3** in the methanol extract of *P. curviflorus* by a validated HPTLC method.

## 2. Materials and Methods

### 2.1. Phytochemical and Spectroscopic Procedures

All the spectroscopic techniques used were the same as reported earlier [11].

### 2.2. Plant Materials

The aerial parts of *P. curviflorus* were collected from Hijaz, Saudi Arabia, and identified by a plant taxonomist of Pharmacy College, King Saud University. A voucher specimen (No. 127) has been placed in the herbarium of Pharmacognosy Department, King Saud University.

#### 2.2.1. Extraction and Phytochemical Isolation from *P. curviflorus*

The aerial parts of the plant (500 g) were ground after shade-dried and successfully extracted with methanol (2 × 1.5 L) at room temperature. Isolation of compounds **P1**, **P2**, and **P3** has been described in our previous published article on the plant [11].

### 2.3. HPTLC Instrumentation and Conditions

The HPTLC analyses of compounds **P1**, **P2**, and **P3** in *P. curviflorus* methanol extract (PCME) were performed on (20 × 10 cm) precoated silica gel *F*_254_ HPTLC plates. During the experiment, the microliter syringe adjusted with the Automatic TLC Sampler-4 was used for spotting the samples and the extract on a HPTLC plate at a rate of 160 nL/sec. The plate was developed under certain condition using GAMAC Automated Developing Chamber-2 (ADC-2). In order to improve resolution and separation of different compounds existing in *P. curviflorus*, a number of trials for best mobile phases combinations were carried out. We found that dichloromethane : methanol : formic acid in the ratio of 90 : 9.5 : 0.5 (v/v/v) is the appropriate mobile phase mixture which was chosen to carry out the analysis. The following chamber saturation condition (at 25 ± 2°C and 60 ± 5% humidity) using a twin-trough glass chamber (20 × 10 cm) was prepared for the development of the plate. Then, the developed plate was dried and scanned by GAMAG TLC Scanner-3 at *λ* = 460 nm (wavelength) in the mode of absorbance.

### 2.4. Preparation of Stock Solutions (Standards)

Seven different concentrations (10, 20, 40, 60, 80, 100, and 120 *µ*g/mL) were prepared from the serial dilution of each standard **P1**, **P2**, and **P3** stock solutions (1 mg/mL) dissolved in methanol. In order to get a linearity range of 100–1200 ng/band, 10 *μ*L of each concentration of **P1**, **P2**, and **P3** were spotted on the HPTLC plate.

### 2.5. Validation of the Method

The projected HPTLC method validation was prepared according to the ICH guideline (ICH, 2005) for LOD (limit of detection), LOQ (limit of quantification), precision, linearity range, robustness, recovery, and accuracy.

#### 2.5.1. Accuracy

The standard addition method was used to determine accuracy. Compounds 1–3 (200 ng/band) were spiked with the extra 0, 50, 100, and 150% of P1, P2, and P3, and the solutions were reanalyzed in six replicates (n = 6) by the proposed method. The percent relative standard deviation (%RSD) and recovery took place.

#### 2.5.2. Precision

Three different concentration values 400, 600, and 800 ng/band of compounds **P1**, **P2**, and **P3** were prepared by duplicate analyses (*n*=6) for calculation of interday and intraday precision of the proposed method. Intraday assays were repeated on three different days for determination of the interday precision.

#### 2.5.3. Robustness

Small deliberate changes were made to the mobile phase composition, mobile phase volume, and duration of mobile phase saturation for analyzing robustness.

The results were determined in terms of %RSD and SD of peak areas after three times repetition of the experiment at 300 ng/band. Different amounts of dichloromethane : methanol : formic acid (v/v/v) were prepared for the chromatography mobile phase. Activation of plates took place for 30 minutes at 110°C before chromatography process.

#### 2.5.4. LOQ and LOD

The slope (*S*) of the calibration curve and the standard deviation (SD) of the response are parameters used to identify the LOQ and the LOD according to the formula: LOQ = 10 (SD/*S*) and LOD = 3.3 (SD/*S*). Taking into consideration that LOQ is the lowest amount that can be determined at a distinct level of accuracy or precision and the LOD is the minimum amount of an analyte that may be calculated from the assay background at a distinct level of confidence.

#### 2.5.5. Assay of Compounds **P1**, **P2**, and **P3**

Compounds **P1**, **P2**, and **P3** and PCME were applied on HPTLC plates. The percentage of the three compounds present in PCME was identified by calculating the area for the markers in PCME.

#### 2.5.6. Statistical Analysis

The statistical analysis was used to determine the total variation in a set of data. Two methods were occupied for this purpose: Dunnet's test and one-way analysis of variance (ANOVA). The achieved results were indicated as mean ± SD; differences were considered significant at *P* values <0.05.

### 2.6. Biological Study

#### 2.6.1. PPAR*α* and PPAR*γ* Agonistic Activity

A reporter gene assay was carried out as described previously [13]. Briefly, upon confluency, the different concentrations of the test samples were exposed to transfected cells for 24 hrs. Vehicle control was taken into consideration when the fold induction in luciferase activity was calculated. Both rosiglitazone and ciprofibrate were used as control drugs for *PPARα* and *PPARγ*, respectively.

#### 2.6.2. Assay for Antioxidant Activity

Human hepatoma cell line (HepG2) was the cell line of choice in this study according to the previously described method [14]. At a density of 60,000 cells/well, the HepG2 cells were seeded in a 96-well plate and incubated for 24 h for confluency. PBS was used for washing the cells, followed by samples treatment. The SpectraMax plate reader was used for reading the plates after addition of 600 *μ*M of ABAP. The area under the curve (AUC) of fluorescence units and time were two parameters used for indication of the percent decrease in oxidative stress. Percent decrease in oxidative stress = 100 − ((AUC sample/AUC control) × 100). Quercetin was used as a positive control.

#### 2.6.3. Assay for iNOS Inhibition

Mouse macrophage (RAW264.7) cells were seeded in the wells of 96-well plates followed by 24 hrs for confluency according to the method described previously [13, 14]. The test samples were treated with LPS (5 *μ*g/mL) and followed by 24-hour incubation as well. The nitrite level in cell supernatant was measured by Griess reagent. The dose response curves gave indication for IC_50_ values. Parthenolide was used as positive control.

#### 2.6.4. Reporter Gene Assay for Inhibition of NF-κB Activity

According to the method described earlier [12, 13], the 96-well plates with density of 1.25 × 10^5^ cells per well were seeded with human chondrosarcoma cells (SW1353) transfected with NF-κB luciferase plasmid construct. After incubation for 24 h, cells were treated with the test samples for half an hour followed by PMA (70 ng/mL) for 8 h. Luciferase assay kit (Promega, Madison, WI, USA) was used for determination of luciferase activity, and parthenolide was used as the positive control in this test.

#### 2.6.5. Assay for Cytotoxicity

Four human cancer cell lines (SK-OV-3, SK-MEL, BT-549 and KB) and two noncancerous kidney cell lines (VERO and LLC-PK1) were measured in the *in vitro* cytotoxic study. Test samples were added on the cell lines with variable concentrations, followed by 48 h incubation. According to the Borenfreund method, the cell viability was determined at the end of incubation [15], using doxorubicin as a positive control.

#### 2.6.6. Antimicrobial Assay

The antimicrobial activity of PCME and isolated compounds **P1**, **P2**, and **P3** was assessed against Gram-negative *Enterobacter xiangfangensis* (CP017183.1), *Escherichia fergusonii* (CU928158.2), and *Pseudomonas aeruginosa* (NR-117678.1) and Gram-positive *Staphylococcus aureus* (CP011526.1) and *Bacillus licheniformis* (KX785171.1) using well diffusion and broth microdilution techniques as described by Berghe and Vlietinck [16]. The bacterial strains were suspended in a nutrient broth for 24 hr, and the suspension then spread on Mueller-Hinton agar. Wells were loaded with 10 *µ*L of the PCME, **P1**, **P2**, and **P3**. Amikacin was used as the positive control. The clear area which was free of microbial growth was measured to detect the diameter of the zone of inhibition (mean of triplicates ± SD), and the least concentration of the PCME and **P1**, **P2**, and **P3** that did not show any visible growth of the bacteria in broth culture (MIC) was calculated as well.

## 3. Results and Discussion

### 3.1. Biological Evaluation of PCME and **P1**, **P2**, and **P3**

Genus *Plicosepalus* comprises two species in Saudi Arabia. Previous work on this genus reported antiviral, antidiabetic, and cytotoxic activities. Little phytochemical and biological attention was drawn on the plant, and few previous studies detected only the hypoglycemic and cytotoxic activities of *P. curviflorus* extract and compounds [11, 17]. In this study, the three compounds **P1**, **P2**, and **P3** were obtained from PCME after subjecting it to a series of silica gel and Sephadex LH-20 column chromatographic separations [11]. The PCME and isolated compounds **P1**, **P2**, and **P3** were subjected to different biological investigations for concluding the complete information about its active constitutes.

Fold induction in the PPAR activity was detected in response to the methanol fraction and pure compounds according to untreated controls. A fold induction of 1.5 means a 50% increase in PPAR activity. Dual activation effect (a fold induction of 1.5 or more) was exhibited by PCME and **P2** (Table 1). Several studies have been carried out with the aim of exploring the potential of selective PPAR*γ* modulators [18]. The dual activator effect of **P2** is in agreement with previous literature [19].

The other two isolates, namely, **P1** and **P3**, showed no PPAR agonistic activity. However, this is the first report of the PPAR agonistic activity of the PCME and **P2**.

From the results shown in Table 2, it could be seen that the PCME, **P1**, **P2**, and **P3** showed decrease in oxidative stress. The largest decrease in oxidative stress was exhibited by the PCME (66% decrease at 500 *µ*g/mL), which may be attributed to its constituents (mainly the flavane gallates and flavonoids). All three constituents **P1**, **P2**, and **P3** showed decrease in oxidative stress by 60%, 52%, and 57%, respectively, at 250 *µ*g/mL. This indicates that the significant antioxidant effect of the PCME seems to be due to its high phenolic content. Flavonoids are polyphenolic substances with free-radical scavenging properties that can inhibit oxidative enzymes and demonstrate anti-inflammatory action [3].

The other targets related to anti-inflammatory activity were only weakly to moderately affected by the PCME, such as that shown by inhibition of the nuclear transcription factor NF-kB and inducible nitric oxide synthase (iNOS) (Table 2). Interestingly, **P2** showed iNOS inhibition while **P3** did not show any. **P1** was the most active iNOS inhabitant with IC_50_ of 25 *µ*g/mL.

All tested compounds **P1**, **P2**, and **P3** from the *P. curviflorus* were not cytotoxic although PCME showed weak activity against all four cancerous cell lines as well as the two normal cell lines with the IC_50_ values ranging from 70–92 *μ*g/mL (Table 3). This may refer to the synergistic activity of polyphenolic constituents present among them.

Several studies have reported that flavonoids and flavane gallates suppress the activity of the pathogenic microorganisms [20–22]. In this study, the PCME and **P1** showed significant antibacterial effect against both Gram-positive and Gram-negative bacteria as compared to amikacin (Table 4), the reference standard. The highest activity was recorded for **P1** against both *Klebsiella pneumonia* (ATCC 700603) and *Pseudomonas aeruginosa* (ATCC 27853) with MIC of 6.2 and 6.6 *µ*g/mL, respectively, followed by the activity of PCME against *Bacillus cereus* (ATCC 14579) and *Pseudomonas aeruginosa* (ATCC 27853) with MIC of 7.6 and 8.4 *µ*g/mL, respectively, while **P2** showed the lowest activity and **P3** showed any noticeable inhibition.

### 3.2. HPTLC Method Development and Validation

**Figure 2:**
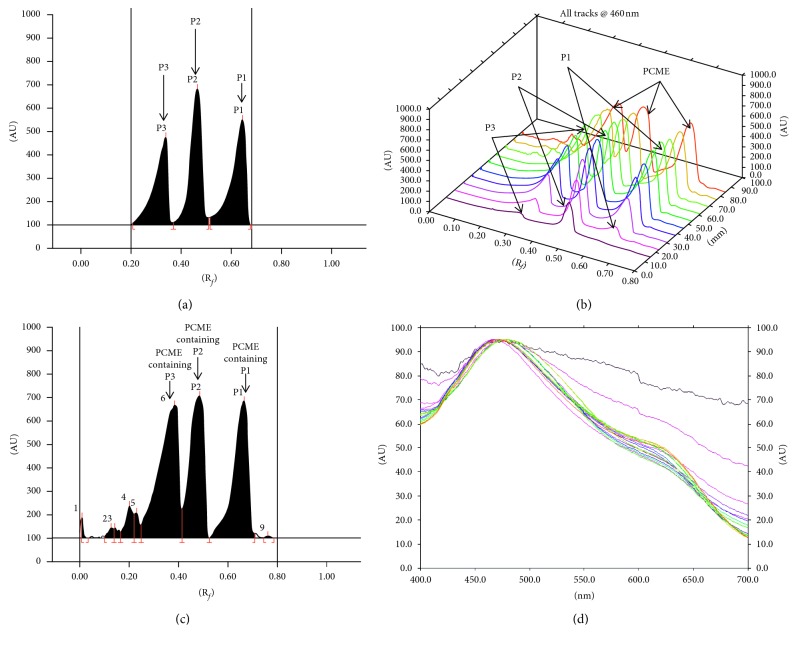
Quantification of **P1**, **P2**, and **P3** in PCME by HPTLC at *λ* = 460 nm (mobile phase: dichloromethane : methanol : formic acid (90 : 9.5 : 0.5)). (a) Chromatogram of standard **P3** (*R*_f_ = 0.34), **P2** (*R*_f_ = 0.47), and **P1** (*R*_f_ = 0.65). (b) 3D display of all tracks. (c) Chromatogram of PCME (**P3**, spot 6, *R*_f_ = 0.34; **P2**, spot 7, *R*_f_ = 0.47; **P1**, spot 8, *R*_f_ = 0.65). (d) Spectral comparison of all the tracks at 460 nm.

Different solvent compositions were analyzed in order to choose the appropriate mobile phase for HPTLC analysis. Out of these, a mixture of dichloromethane : methanol : formic acid in the ratio of 90 : 9.5 : 0.5 (v/v/v) was found to be the most effective mobile phase for the estimation **P1**, **P2**,and **P3**. Sharp and compact peaks of **P1**, **P2**, and **P3** took place at *R*_f_ = 0.34, 0.47, and 0.65, respectively (Figure 2(a)), with good separation of the biomarkers from the matrix and other several constituents of the PCME (Figure 2(b)). 20 mL and 20 min were the optimized mobile phase volume for saturation and saturation time, respectively. High-resolution baseline affects the selectivity of our developed method. The identities of the bands of the extracts were confirmed by overlaying their spectra along with the spectra of standards (Figure 2(d)). The regression equation/square of correlation coefficients (*r*^2^) for **P1**, **P2**, and **P3** were found as *Y* = 2.725*X* + 122.44/0.993, *Y* = 1.675*X* + 755.87/0.993, and *Y* = 52.008*X* + 211.51/0.9972, respectively, in the linearity range 100–1200 ng/spot. The limit of detection (LOD) and limit of quantification (LOQ) were found as 31.04/94.06, 29.23/88.58, and 12.33/37.36, respectively, for **P1**, **P2**, and **P3** (Table 5). The accuracy values of the proposed method for estimation of P1, P2, and P3 were observed in terms of % recovery and %RSD, as listed in (Table 6). The recovery/RSD (%) for **P1**, **P2**, and **P3** was found as 98.89–99.72/1.25–1.35, 98.57–99.07/1.41–1.52, 98.36*-*99.22/1.21–1.36, respectively (Table 5). The %RSD for intraday/interday precisions (*n*=6) were detected as 1.15–1.20/1.11–1.20, 1.17–1.27/1.12–1.21, and 1.27–1.33/1.271.32 respectively, for **P1**, **P2**, and **P3** which exhibited good precision of the proposed method (Table 7). The robustness study was determined by introducing little deliberate changes in the mobile phase constituent, saturation duration time, and the volume of mobile phase, and the recorded data in the form of SD and %RSD values are listed in Table 8. The low values of SD and %RSD showed that the proposed method was robust.

### 3.3. Estimation of **P1**, **P2**, and **P3** in PCME by HPTLC

The developed HPTLC technique was used for quantitative analysis of **P1**, **P2**, and **P3** in PCME. These biologically active markers were found to be present in PCME (Figure 2(c)). The quantities (*μ*g/mg of the dried weight of extracts) of biomarkers **P1**, **P2**, and **P3** in PCME were found in the following order: 11.06 > 10.9 > 6.96 *μ*g/mg (Table 9). This finding indicated that **P1** is the highest quantity of all the biomarkers followed by **P2** and **P3** in the PCME.

Different analytical techniques such as HPLC, HPTLC had been reported for the quantitative analysis of quercetin in many plant extracts such as detection of quercetin by the HPTLC method in the aerial parts of *Leea indica* [23] and *Thespesia populnea* L. [24]. To our knowledge, this is the first report of concurrent estimation of quercetin (**P1**), 2S,3R-3,3′,4′,5,7-pentahydroxyflavane-5-O-gallate (**P3**), and (-)-catechin (**P2**) in *P. curviflorus*. Hence, we are privileged to report this maiden research on the screening of antidiabetic, cardiovascular, anti-inflammatory, and cytotoxic biomarkers in the methanol extract of the plant.

## 4. Conclusion

Compounds **P1**, **P2**, and **P3** have been isolated from the methanolic extract of the leaves of *P. curviflorus*. All compounds and the extract showed significant antioxidant activities. Catechin (**P2**) and the methanol extract (PCME) revealed dual PPAR activation effect, while compound **P1** possesses the most potential anti-inflammatory activity. The maiden HPTLC method was developed for the simultaneous analysis of the biomarker compounds quercetin (**P1**), (-)-catechin (**P2**), and 2S,3R-3,3′,4′,5,7-pentahydroxyflavane-5-O-gallate (**P3**) in the methanol extract of *P. curviflorus* (PCME). The proposed HPTLC method can be further applied for the analysis of these compounds **P1**, **P2**, and **P3** in different plant extracts, biological samples, and herbal formulations as well as for the in-process quality control.

## Figures and Tables

**Table 1:** PPAR agonistic activity of PCME and **P1**, **P2**, and **P3**.

Sample name	Fold induction					
	PPAR alpha			PPAR gamma		
	50 *µ*g/mL	25 *µ*g/mL	12.5 *µ*g/mL	50 *µ*g/mL	25 *µ*g/mL	12.5 *µ*g/mL
PCME	1.50	1.31	1.21	1.90	1.90	1.60
**P1**	NA	NA	NA	NA	NA	NA
**P2**	1.47	1.31	1.19	1.53	1.55	1.19
**P3**	NA	NA	NA	NA	NA	NA
Ciprofibrate (10 *µ*M)	2.2			NT		
Rosiglitazone (10 *µ*M)	NT			3.7		

NA = not active.

**Table 2:** Anti-inflammatory activity of PCME and **P1**, **P2**, and **P3**.

^a^Sample name	% decrease in oxidative stress	iNOS inhibition (IC_50_; *µ*g/mL)	NF-kB inhibition (IC_50_; *µ*g/mL)
PCME	66	NA	NA
**P1**	52	48	NA
**P2**	39	>50	NA
**P3**	57	NA	NA
Quercetin (50 *µ*M)^b^	75	—	—
Parthenolide^b^	—	0.35 ± 0.22	0.5 ± 0.21

^a^At 500 *µ*g/mL for the extract and 250 *µ*g/mL for pure compounds. ^b^Positive control. NA = no activity up to 100 *µ*g/mL (NF-kB, iNOS) and 500 *µ*g/mL (oxidative stress).

**Table 3:** Cytotoxicity of PCME and **P1**, **P2**, and **P3**.

Sample name	Cancer cells (IC_50_; *µ*g/mL)				Noncancer cells (IC_50_; *µ*g/mL)	
	SK-MEL	KB	BT-549	SK-OV-3	VERO	LLC-PK11
PCME	76	60	92	70	70	70
**P1**	NA	NA	NA	NA	NA	55
**P2**	NA	NA	NA	NA	NA	16
**P3**	NA	NA	NA	NA	NA	NA
Doxorubicin	1.23	1.85	1.93	0.8	>5	0.85

NA = no cytotoxic activity upto 100 *µ*g/mL.

**Table 4:** Antibacterial activity of PCME and **P1**, **P2**, and **P3**.

Bacterial strain	ZOI diameter (mm)					MIC (*µ*g/mL)				
	PCME	**P1**	**P2**	**P3**	AMK	PCME	**P1**	**P2**	**P3**	AMK
Gram −										
*K. pneumonia*	13 ± 1.8	21.3 ± 1.15	—	—	25 ± 0.8	10	6.2	—	—	5
*E. fergusonii*	17 ± 0.5	16.3 ± 0.57	8 ± 1	—	24 ± 1.5	8.9	9.3	11	—	4.5
*P. aeruginosa*	18 ± 1.2	21 ± 1	—	—	18 ± 1.3	8.4	6.6	—	—	8
Gram +										
*S. aureus*	16 ± 1.6	18 ± 1	—	—	24 ± 1	9.2	8.3	—	—	6.2
*B. cereus*	20 ± 0.5	19.3 ± 1.15	—	—	20 ± 0.9	7.6	8.4	—	—	7.3

Results are expressed as mean ± standard deviation (SD).

**Table 5:** *R*_f_ and linear regression data for the calibration curve of **P1**, **P2**, and **P3** (*n* = 6).

Parameters	**P1**	**P2**	**P3**
Linearity range (ng/spot)	100–1200	100–1200	100–1200
Regression equation	*Y* = 2.725*X* + 122.44	*Y* = 1.675*X* + 755.87	*Y* = 2.008*X* + 211.51
Correlation (*r*^2^) coefficient	0.993 ± 0.001	0.993 ± 0.001	0.9972 ± 0.001
Slope ± SD	2.725 ± 0.025	1.675 ± 0.014	2.008 ± 0.007
Intercept ± SD	122.44 ± 9.30	755.87 ± 10.07	211.51 ± 12.75
Standard error of slope	0.005	0.006	0.003
Standard error of intercept	3.795	4.11	5.206
*R*_f_	0.65 ± 0.004	0.47 ± 0.003	0.34 ± 0.001
LOD (ng)	31.04	29.23	12.33
LOQ (ng)	94.06	88.58	37.36

**Table 6:** Recovery as accuracy studies of the proposed HPTLC method (*n* = 6).

Percent of **P1**, **P2**, and **P3** added to analyte	Theoretical concentration of **P1**, **P2**, and **P3** (ng/band)	**P1**			**P2**			**P3**		
		Concentration found (ng/band) ± SD	%RSD	% recovery	Concentration found (ng/band) ± SD	%RSD	% recovery	Concentration found (ng/band) ± SD	%RSD	% recovery
0	200	197.78 ± 2.57	1.29	98.89	198.14 ± 2.79	1.41	99.07	196.72 ± 2.38	1.21	98.36
50	300	296.07 ± 3.72	1.25	98.69	296.65 ± 4.37	1.47	98.88	297.66 ± 3.67	1.23	99.22
100	400	398.88 ± 5.31	1.33	99.72	396.25 ± 5.91	1.49	99.06	396.17 ± 5.29	1.33	99.04
150	500	495.02 ± 6.73	1.35	99.01	492.85 ± 7.54	1.52	98.57	494.28 ± 6.73	1.36	98.85

**Table 7:** Precision of the proposed HPTLC method (*n* = 6).

Conc. of standard added (ng/band)	**P1**				**P2**				**P3**			
	Intraday precision		Interday precision		Intraday precision		Interday precision		Intraday precision		Interday precision	
	Average conc. found ± SD	%RSD	Average conc. found ± SD	%RSD	Average conc. found ± SD	%RSD	Average conc. found ±SD	%RSD	Average conc. found ± SD	%RSD	Average conc. found ± SD	%RSD
200	197.78 ± 2.29	1.15	194.47 ± 2.17	1.11	198.02 ± 2.33	1.17	196.23 ± 2.21	1.12	198.12 ± 2.53	1.27	193.14 ± 2.47	1.27
400	395.94 ± 4.77	1.20	392.27 ± 4.63	1.18	396.45 ± 4.77	1.20	392.87 ± 4.59	1.16	396.31 ± 5.21	1.31	393.32 ± 5.19	1.31
600	597.81 ± 7.17	1.19	594.14 ± 7.15	1.20	594.10 ± 7.59	1.27	591.72 ± 7.17	1.21	594.75 ± 7.95	1.33	592.26 ± 7.85	1.32

**Table 8:** Robustness of the proposed HPTLC method (*n* = 6).

Optimization condition	**P1**		**P2**		**P3**	
	SD	%RSD	SD	%RSD	SD	%RSD
Mobile phase composition (dichloromethane : methanol : FA = 90 : 9.5 : 0.5)						
90 : 9.5 : 0.5	3.51	1.18	3.31	1.12	4.26	1.42
89.5 : 10 : 0.5	3.57	1.19	3.37	1.15	4.29	1.44
90.5 : 9 : 0.5	3.62	1.21	3.41	1.18	4.37	1.46
Mobile phase volume (for saturation)						
18 mL	3.34	1.13	3.62	1.23	4.17	1.40
20 mL	3.38	1.14	3.67	1.25	4.23	1.43
22 mL	3.42	1.16	3.69	1.27	4.31	1.45
Duration of saturation						
10 min	3.45	1.16	3.21	1.08	4.01	1.35
20 min	3.49	1.18	3.26	1.09	4.03	1.36
30 min	3.52	1.19	3.31	1.10	4.09	1.38

**Table 9:** HPTLC analysis of **P1**, **P2**, and **P3** in PCME.

S. No.	Sample	**P1** content (*µ*g/mg of dried weight of extract)	**P2** content (*µ*g/mg of dried weight of extract)	**P3** content (*µ*g/mg of dried weight of extract)
1	PCME	11.06 ± 0.86	10.9 ± 0.77	6.96 ± 0.41

## Data Availability

The data used to support the findings of this study are available from the corresponding author upon request.
